# Adherence to the EAT-Lancet diet and the risk of heart failure in individuals with cardiovascular-kidney-metabolic syndrome stages 0–3: A prospective cohort study

**DOI:** 10.3389/fnut.2026.1802388

**Published:** 2026-05-13

**Authors:** Peng Fu, Hong Zheng, Kuangyi Wu, Yulong Lan, Dan Wu, Weiqiang Wu, Huancong Zheng, Zefeng Cai, Yuxian Wang, Qiuyue Lin, Bo Zhang, Youren Chen

**Affiliations:** 1 Department of Cardiology, Second Affiliated Hospital of Shantou University Medical College, Shantou, Guangdong, China; 2 Shantou University Medical College, Shantou, Guangdong, China; 3 School of Medical and Health Sciences, Edith Cowan University, Joondalup, WA, Australia; 4 Institute of Cardiovascular Diseases, First Affiliated Hospital of Dalian Medical University, Dalian, China; 5 Department of Cardiology, The First Affiliated Hospital of Dalian Medical University, Dalian, Liaoning, China

**Keywords:** cardiovascular diseases, cardiovascular kidney metabolic syndrome, EAT-Lancet diet, heart failure, plant-based diet

## Abstract

**Background and objective:**

In 2019, the EAT-Lancet Commission on Healthy Diets from Sustainable Food Systems proposed the EAT-Lancet planetary health diet as a universal reference dietary pattern aimed at promoting human health while minimizing environmental degradation. However, epidemiological evidence regarding the association between adherence to the EAT-Lancet diet and the risk of heart failure (HF), particularly among individuals with cardiovascular-kidney-metabolic syndrome (CKMs) stages 0–3, remains limited. Therefore, this study aimed to examine the associations between adherence to the EAT-Lancet dietary pattern, its individual dietary components, and the risk of HF among individuals at CKMs stages 0–3.

**Materials and methods:**

In this prospective study, a total of 120,849 participants with CKMs stages 0–3 from the UK Biobank cohort were included. Dietary data were collected using an online 24-h dietary recall questionnaire. The EAT-Lancet Diet Index, ranging from 0 to 14, was constructed based on the EAT-Lancet reference diet to evaluate adherence. Cox proportional hazards models and restricted cubic spline functions were used to assess the associations between the EAT-Lancet Diet Index, its dietary components, and HF risk.

**Results:**

Over a median follow-up of 14.88 years, 2,454 participants developed HF. A higher EAT-Lancet Diet Index was significantly associated with a lower risk of HF compared to the lowest index group (hazard ratio [HR]: 0.88; 95% confidence interval [CI]: 0.80, 0.97). This association remained robust across subgroup and multiple sensitivity analyses. Restricted cubic spline analysis revealed a significant inverse linear relationship between the EAT-Lancet Diet Index and HF risk. Among dietary components, reduced intake of red meat (beef, lamb, and pork) (HR: 0.90; 95% CI: 0.83, 0.98) and increased intake of peanuts or other nuts (HR: 0.76; 95% CI: 0.60, 0.95) were significantly associated with lower HF risk.

**Conclusion:**

This study demonstrates that greater adherence to the EAT-Lancet diet is significantly associated with a reduced relative hazard of HF among individuals with CKM stages 0–3, particularly through reduced consumption of red meat and increased intake of peanuts or other nuts.

## Graphical abstract

**Figure:**
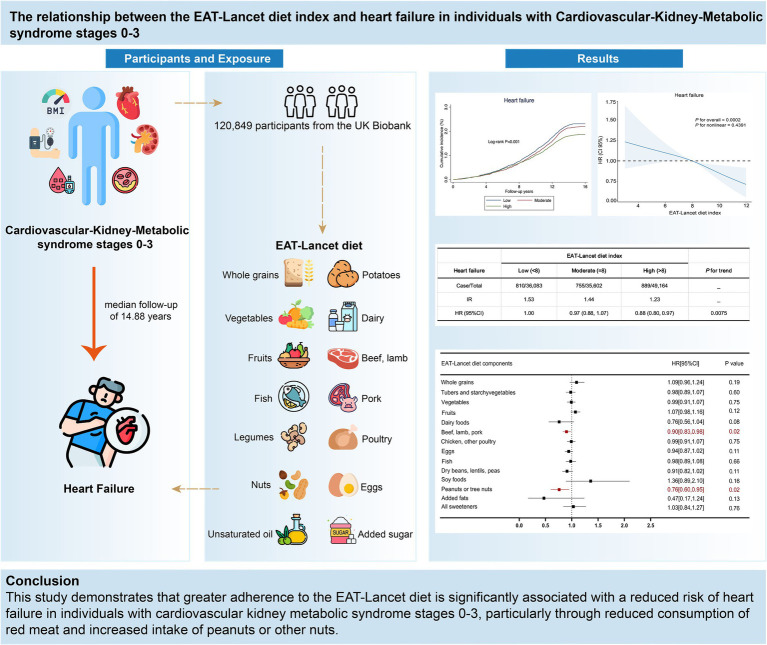

## Introduction

Cardiovascular-Kidney-Metabolic Syndrome (CKMs) was first introduced by the American Heart Association (AHA) (1). It is characterized by the synergistic dysfunction of cardiovascular diseases (CVD), chronic kidney disease, and metabolic system disturbances, and is classified into five stages, from stage 0 (no CKM risk factors) to stage 4 (clinical CVD). Compared to stages 0–3, individuals in stage 4 have a significantly higher risk of adverse cardiovascular events and mortality (1–5). Therefore, the AHA recommends early intervention in individuals with CKMs stages 0–3 (6), aiming to capture the critical window for prevention to slow the progression of CKMs and improve adverse outcomes.

In this context, heart failure (HF), as a late-stage manifestation of many CVD, has become one of the major health challenges faced by individuals with CKMs. In 2017, approximately 64 million people worldwide had HF, and its prevalence is increasing at an alarming rate due to the growing elderly population (7). Despite significant advances in treatment that have notably improved patient prognosis, the incidence and mortality of HF remain high (8), imposing a substantial burden on both society and healthcare systems (7). Importantly, individuals with CKMs stages 0–3 often exhibit subclinical metabolic dysfunction, low-grade inflammation, insulin resistance, and hypertension—pathophysiological processes that are central to the development of HF (9). Therefore, identifying modifiable lifestyle factors during these early CKMs stages is essential for preventing HF onset and improving long-term cardiovascular outcomes.

Dietary patterns represent one of the most important and modifiable lifestyle factors influencing cardiometabolic health. Accumulating evidence suggests that balanced, plant-forward dietary patterns can favorably affect key pathways involved in HF development, including systemic inflammation, oxidative stress, endothelial dysfunction, blood pressure regulation, and metabolic homeostasis (10, 11). The EAT-Lancet diet, proposed by the EAT-Lancet Commission in 2019 (12), also known as the “Planetary Health Diet,” emphasizes a plant-based approach to healthy eating. This dietary pattern recommends a focus on whole grains, vegetables, fruits, legumes, and nuts, while limiting the intake of red meat, processed foods, and sugars (13). Existing studies have shown that adherence to the EAT-Lancet diet can effectively improve cardiovascular health (11), reduce inflammation levels, and lower the incidence of metabolic diseases (14, 15). However, research on the relationship between the EAT-Lancet diet and HF risk in individuals with CKMs stages 0–3 is still lacking.

Therefore, using data from the UK Biobank, a large prospective cohort study with comprehensive dietary information, we aim to explore the association between adherence to the EAT-Lancet diet and the risk of HF in individuals with CKMs stages 0–3.

## Methods

### Study population

The UK Biobank is a prospective cohort study that recruited 500,000 participants aged 37 to 73 years from 21 assessment centers across England, Wales, and Scotland between 2006 and 2010. Participants provided comprehensive health-related information through questionnaires, including demographic data, medical history, medication use, smoking and drinking habits, and detailed dietary records. They also underwent physical examinations and provided blood samples at baseline. The study design and data collection have been described in previous literature (16, 17).

**Figure 1:**
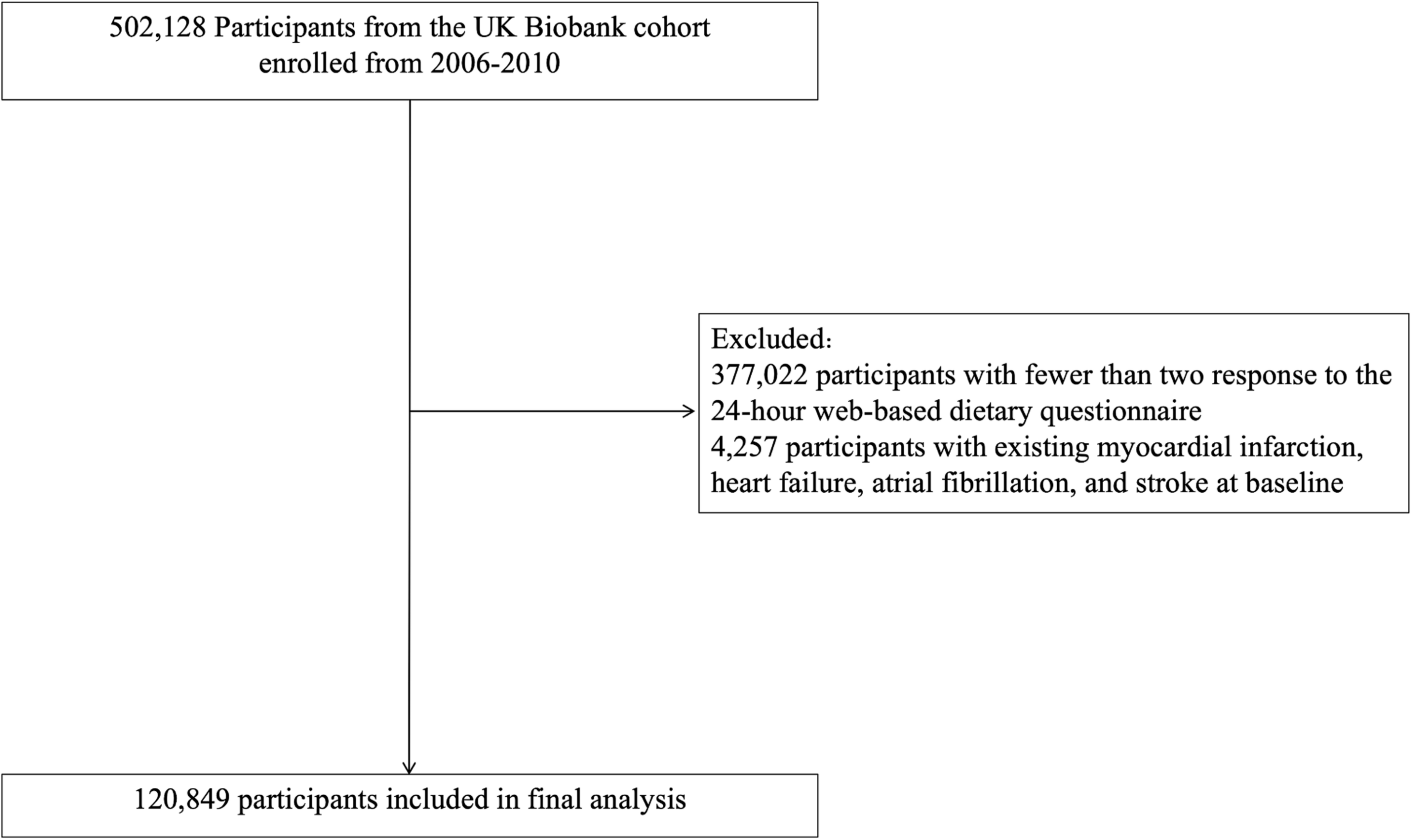
Study flowchart.

The cohort was followed from the baseline survey date until the study end date (May 31, 2024). Participants who answered the 24-h online dietary questionnaire less than twice (*n* = 377,022) and those with a history of CVD (*n* = 4,257), including myocardial infarction, HF, atrial fibrillation, and stroke, were excluded. The final analysis included 120,849 participants (Figure 1). All participants in the UK Biobank study provided informed consent. The use of data in this study was approved by the Northwest Multi-Center Research Ethics Committee. This study complies with the UK Biobank data access procedures and was authorized under application number 156009.

### Definition of CKMs stages 0–3

The staging of CKMs stages 0–3 is defined based on the scientific statement from the AHA.

(1) Stage 0 is defined as the absence of CKM health risk factors, including overweight or obesity, metabolic abnormalities (hypertension, hypertriglyceridemia, metabolic syndrome, and diabetes), chronic kidney disease (CKD), and subclinical or clinical CVD.
(2) Stage 1 represents excessive and/or dysfunctional obesity, identified by increased waist circumference (WC), body mass index (BMI), or impaired fasting glucose/hemoglobin A1c.
(3) Stage 2 includes individuals with metabolic risk factors and/or CKD.
(4) Stage 3 refers to subclinical CVD in the CKMs spectrum, defined as a high 10-year predicted CVD risk.

Detailed definitions or calculations for each stage are provided in the Supplemental material.

### Dietary assessment and EAT-lancet diet index

The dietary questionnaire information used in this study was collected through the Oxford WebQ questionnaire from the UK Biobank (5, 18–20). This questionnaire assessed the types and quantities of foods consumed over a 24-h period. The data were based on participants’ detailed recall of their dietary intake from the previous day, a method that has been widely validated (21). However, not all participants completed the dietary assessment; some participants did not provide any data, while others provided multiple assessments over time. To account for potential biases arising from having only one dietary assessment (20, 22), we included only participants who provided at least two dietary intake records. The average dietary intake was calculated using all available data from these participants (23), to explain dietary pattern differences and enhance the reliability of dietary information.

In this study, we followed the criteria established in previous research, known as the Knuppel score, based on key food components such as whole grains, tubers and starchy vegetables, vegetables, fruits, dairy, protein sources, legumes, added fats, and sugars (21, 24, 25), as shown in Supplementary Table S1. The construction criteria for the EAT-Lancet diet index are outlined in Supplementary Table S2. Briefly, the total score ranges from 0 to 14, with higher scores indicating greater adherence to the EAT-Lancet diet. In this study, participants were divided into three groups based on the median EAT-Lancet diet index (26, 27), with the distribution of the EAT-Lancet diet index shown in Supplementary Figure S1.

### Definition of outcome event

The outcome of interest in this study is the occurrence of HF, identified through hospitalization records with specific ICD-10 codes (I11.0, I13.0, I13.2, I50.0, I50.1, I50.9). Participants were followed from baseline enrollment (2006–2010) until the occurrence of HF, death, or the end of the follow-up period (May 31, 2024), whichever occurred first.

### Assessment of covariates

Covariate information was collected at baseline through a touchscreen questionnaire. Age and sex were self-reported. Ethnicity was categorized as White or Other. The Townsend Deprivation Index (TDI), which includes factors such as unemployment, overcrowding, lack of resources, and housing insecurity, was used to assess socioeconomic status, with higher scores indicating greater social deprivation. Education level was categorized as whether the participant held a university degree. Household annual income was divided into four groups: below £18,000, £18,000–£30,999, £31,000–£51,999, £52,000–£100,000, or above £100,000. Smoking and alcohol use were categorized as never/former or current use. Self-reported family history of CVD was also collected through the baseline questionnaire.

### Statistical analysis

The baseline characteristics of the study participants were described as means ± SD for continuous variables and as frequencies and numbers (percentages) for categorical variables. The distribution of CKMs stages in relation to the EAT-Lancet diet index was visualized using bar charts. To minimize potential bias and retain statistical power, missing values in covariates (≤5%) were addressed using multiple imputation by chained equations (SAS [SAS Institute] PROC MI with a fully conditional specification method and PROC MIANALYZE) (28, 29).

The cumulative incidence of HF risk for each EAT-Lancet diet index group was calculated using the Kaplan–Meier method and compared using the log-rank test. The proportional hazards assumption for the Cox model was assessed using Schoenfeld residuals. After confirming the proportional hazards assumption, Cox proportional hazards models were used to evaluate the associations of the EAT-Lancet diet index and its individual components with HF risk. The EAT-Lancet diet index was analyzed as both a categorical and a continuous variable. For continuous analyses, hazard ratios (HRs) with 95% confidence intervals (CIs) were estimated per SD increase. For analyses of individual dietary components, all components were included simultaneously in the model to assess their independent associations. The results were expressed as HR with 95% CIs. Multivariable models were adjusted for age, sex, ethnicity, alcohol use, smoking status, TDI, income, education, family history of CVD. The EAT-Lancet diet index was treated as an ordinal variable to calculate the *p*-value for trend.

To explore the dose–response relationship between the EAT-Lancet diet index and incident HF among individuals with CKMs stages 0–3, restricted cubic spline models with three knots (at the 10th, 50th, and 90th percentiles of the EAT-Lancet diet index) were employed. The median value of the EAT-Lancet diet index (8.0) was used as the reference point (HR = 1.00).

Subgroup analyses were conducted by stratifying the data based on sex, age (<65 years vs. ≥65 years), education level (university degree vs. below university level), smoking status (non-smoker vs. current smoker), and alcohol consumption (non-drinker vs. current drinker). We performed a series of sensitivity analyses to assess the robustness of the results. We performed a series of sensitivity analyses to assess the robustness of the results. First, participants who experienced outcome events within 2 years of follow-up were excluded to minimize potential reverse causality. Second, participants with incomplete covariate data were excluded to avoid potential bias related to the imputation method. Third, to account for potential competing risks of non-HF mortality (e.g., death from CVD or other causes), we repeated the analyses using the Fine-Gray competing-risk model. Fourth, we restricted the analysis to individuals with CKMs stages 1–3 (excluding those in CKMs stage 0). Fifth, we further adjusted for cardiometabolic variables, including systolic and diastolic blood pressure, fasting blood glucose, estimated glomerular filtration rate, high- and low-density lipoprotein cholesterol, WC, and BMI. BMI and WC were considered as potential mediators, given their established roles in the pathway linking diet to cardiometabolic health and HF risk (30–32). Subsequently, survival mediation analysis was conducted using the *SAS* “mediate” *macro* to examine the mediation effect of BMI and WC in the relationship between the EAT-Lancet diet index and HF. The models were adjusted for age, sex, ethnicity, current drinker, current smoker, TDI, income, education, family history of CVD.

All analyses were performed using *SAS* software (version 9.4) and *R* software (version 4.5.1). A two-tailed *p*-value of <0.05 was considered statistically significant.

## Results

### Baseline characteristics of the study participants

A total of 120,849 participants (43.2% male) were included in the study analysis, with a mean age of 56.0 ± 7.8 years. The baseline characteristics of the study participants are presented in Table 1. The proportions of participants in CKMs stages 0–3 were as follows: stage 0: 23.7%, stage 1: 19.6%, stage 2: 54.6%, and stage 3: 2.1%. Compared with participants in the lowest EAT-Lancet diet index group, those in higher diet index groups were more likely to be female, have higher educational attainment and annual income, and be White. They were less likely to be current drinkers, while current smoking status was comparable across groups. Townsend deprivation index scores were slightly higher in participants with higher EAT-Lancet diet index scores.

**Table 1:** Baseline characteristics of the study participants.

Variables	Total (*N* = 120,849)	Categories of the EAT-Lancet diet index by points			*p* value
		Low (<8) *N* = 36,083	Moderate (=8) *N* = 35,602	High (>8) *N* = 49,164	
Age, years	56.0 ± 7.8	56.4 ± 7.8	56.1 ± 7.8	55.6 ± 7.9	<0.001
Men, (%)	52,214 (43.2%)	16,349 (45.3%)	15,567 (43.7%)	20,298 (41.3%)	<0.001
Race, (%)					<0.001
White	116,975 (96.8%)	35,129 (97.4%)	34,575 (97.1%)	47,271 (96.1%)	
Others	3,874 (3.2%)	954 (2.6%)	1,027 (2.9)	1,839 (3.9)	
Education					
College or university, (%)	61,169 (50.6%)	17,547 (48.6%)	17,772 (49.9%)	25,850 (52.6%)	<0.001
Others	59,680 (49.4%)	18,536 (51.4%)	17,830 (50.1%)	23,314 (47.4%)	
CKMs stages, (%)					<0.001
0	28,663 (23.7%)	7,896 (21.9%)	8,204 (23.0%)	12,563 (25.6%)	
1	23,620 (19.6%)	6,911 (19.2%)	6,957 (19.5%)	9,752 (19.8%)	
2	66,018 (54.6%)	20,425 (56.6%)	19,702 (55.3%)	25,891 (52.7%)	
3	2,548 (2.1%)	851 (2.4%)	739 (2.1%)	958 (1.9%)	
Family history of CVD, (%)	40,326 (33.4%)	12,393 (34.3%)	11,859 (33.3%)	16,074 (32.7%)	0.063
Townsend deprivation index	−1.6 ± 2.8	−1.7 ± 2.8	−1.7 ± 2.8	−1.6 ± 2.9	<0.001
Annual income, £, (%)					<0.001
<18,000	16,381 (13.5%)	4,986 (13.8%)	4,856 (13.6%)	6,539 (13.3%)	
18,000–30,999	28,518 (23.6%)	8,695 (24.1%)	8,477 (23.8%)	11,346 (23.1%)	
31,000–51,999	35,115 (29.1%)	10,523 (29.1%)	10,261 (28.8%)	14,331 (29.1%)	
52,000–100,000	31,296 (25.8%)	9,084 (25.2%)	9,168 (25.8%)	13,044 (26.5%)	
>100,000	9,539 (7.9%)	2,795 (7.7%)	2,840 (8.0%)	3,904 (7.9%)	
Current smoking, (%)	8,389 (6.9%)	2,477 (6.9%)	2,502 (7.0%)	3,410 (6.9%)	0.690
Current drinking, (%)	113,985 (94.3%)	34,201 (94.8%)	33,701 (94.7%)	46,083 (93.7%)	<0.001

Categorical variables are presented as *n* (%), and continuous variables are presented as mean ± SD or median (interquartile range), depending on variable distribution. CKMs, cardiovascular-kidney-metabolic syndrome; CVD, cardiovascular diseases; TDI, townsend deprivation index.

### Association of EAT-lancet diet index with HF events

**Figure 2:**
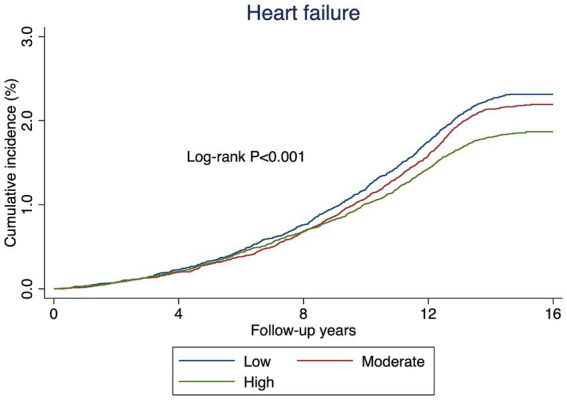
Kaplan–meier curves of incidence of heart failure according to the EAT-Lancet diet index.

**Figure 3:**
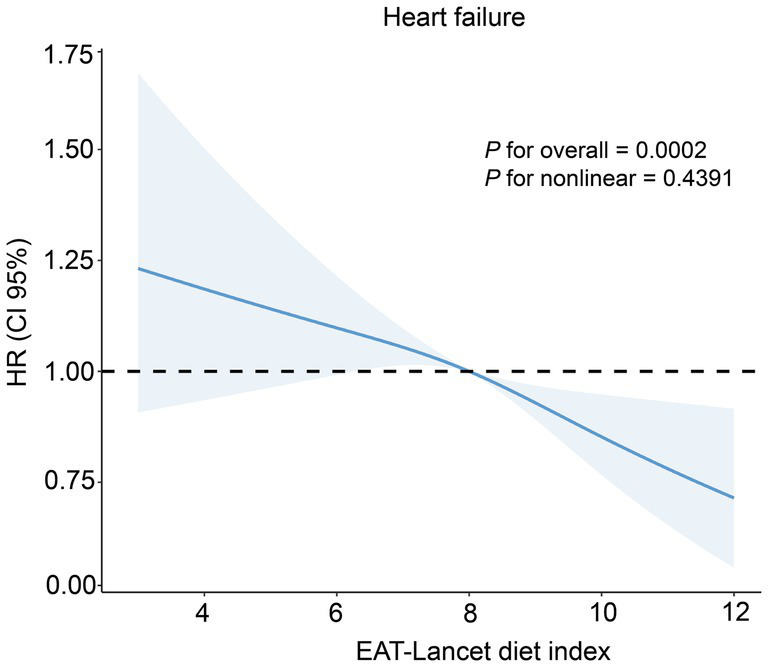
Restricted cubic spline hazard regression plot depicting the relationship between EAT-Lancet diet index and the risk of incident heart failure.

During a median follow-up period of 14.88 years, 2,454 participants experienced HF events. The cumulative incidence curves showed that participants in the higher EAT-Lancet diet index group had a significantly lower risk of HF compared to those in the other groups (Figure 2). Among participants with CKMs stages 0–3, the high EAT-Lancet diet index group was significantly associated with a lower incidence of HF events compared to the low EAT-Lancet diet index group (HR for high EAT-Lancet diet index group: 0.88, 95% CI: 0.80, 0.97) (Table 2). Moreover, when the EAT-Lancet index was modeled as a continuous variable, each 1-SD increase was significantly associated with a lower risk of HF (Table 2). The restricted cubic spline analysis showed a continuous decrease in the risk of incident HF events with increasing EAT-Lancet diet index (Figure 3).

**Table 2:** Association between EAT-Lancet diet index and risk of heart failure outcomes among individuals with CKMs stages 0–3.

Category	Event/total	IR	Unadjusted HR (95% CI)	Adjusted HR (95% CI)	*p* for trend
EAT-Lancet diet index					0.008
Low	810/36,083	1.53	1.00	1.00	–
Moderate	755/35,602	1.44	0.94 (0.85, 1.04)	0.97 (0.88, 1.07)	–
High	889/49,164	1.23	0.80 (0.73, 0.88)	0.88 (0.80, 0.97)	–
Per 1-SD increase	–	–	0.91 (0.88, 0.95)	0.95 (0.91, 0.98)	–

EAT-Lancet index was categorized as low (<8), moderate (=8), and high (>8). The Cox proportional hazards models were adjusted for age, sex, current drinking, current smoking, education, income, Ethnicity, Townsend deprivation index, Family history of CVD.

CKMs, cardiovascular-kidney-metabolic syndrome; IR, incidence rate (per 1,000 person-years); HR, hazard ratio; and CI, confidence interval.

### Association of EAT-lancet diet components with HF events

**Figure 4:**
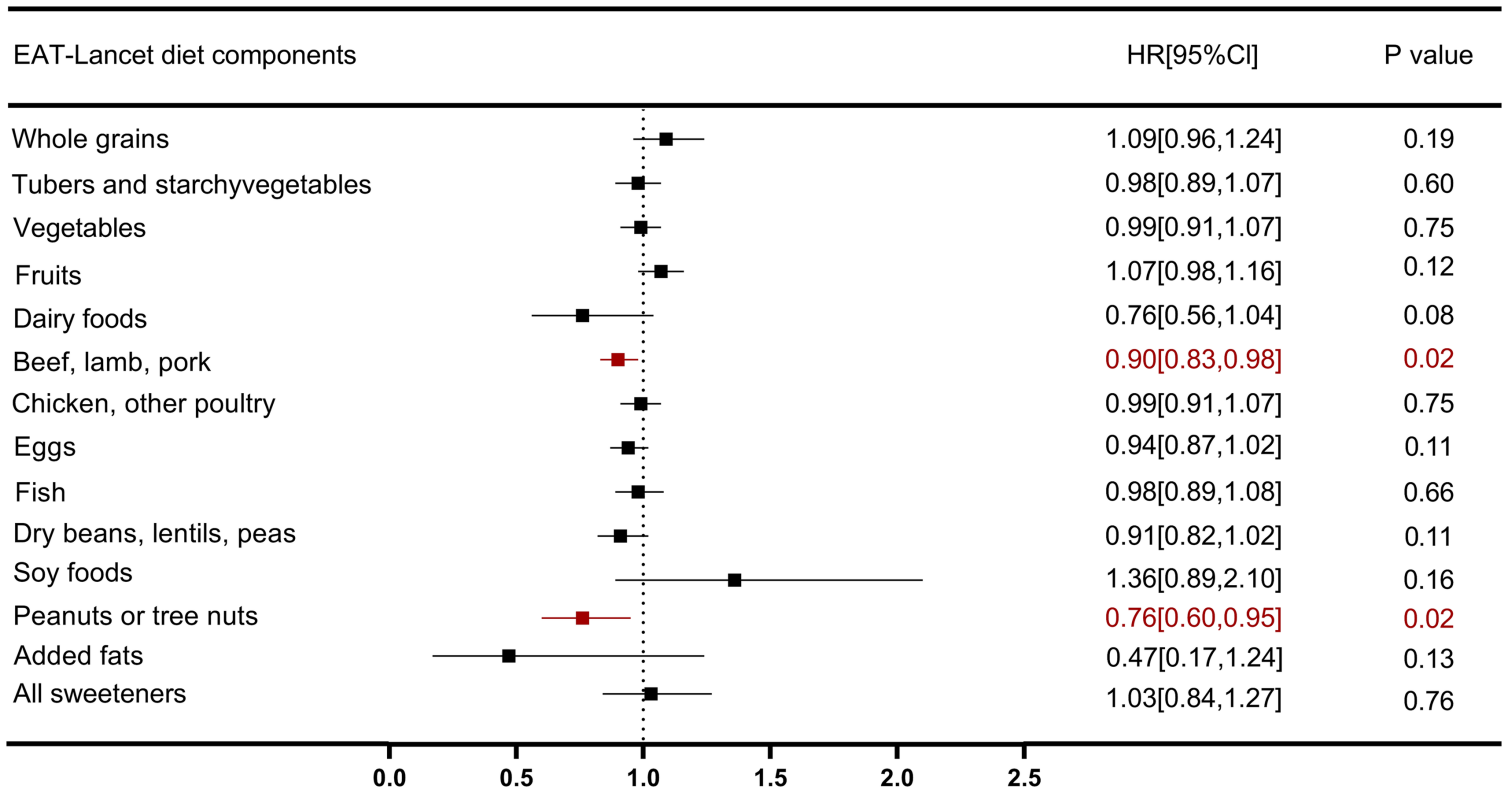
Association between EAT-Lancet diet index components and risk of heart failure outcomes.

Figure 4 illustrates the associations between individual components of the EAT-Lancet diet and HF events. Among all dietary components, reduced intake of red meat (beef, lamb, and pork) (HR: 0.90, 95% CI: 0.83, 0.98) and increased intake of peanuts or nuts (HR: 0.76, 95% CI: 0.60, 0.95) were associated with a lower risk of HF, suggesting that reducing red meat intake and increasing peanuts or nuts intake are significantly inversely related to the risk of HF events, whereas other dietary components were not significantly associated with HF risk.

### Subgroup and sensitivity analyses

As shown in Supplementary Table S3, the results of subgroup analyses stratified by age, sex, education level, current smoker, and current drinker were consistent with the primary analysis. Notably, a significant interaction was observed between the EAT-Lancet diet index and education level (*P*_interaction_ = 0.0487). In the sensitivity analyses, excluding participants who experienced outcome events within the first 2 years of follow-up did not materially alter the results (Supplementary Table S4). Similarly, excluding participants with missing covariate data showed consistent results (Supplementary Table S5). Results were also unchanged when accounting for the competing risk of non-HF mortality using the Fine-Gray model (Supplementary Table S6). Restricting the analysis to participants with CKMs stages 1–3, excluding those in CKMs stage 0, showed similar results (Supplementary Table S7). Further adjustment for cardiometabolic variables also did not materially change the associations (Supplementary Table S8).

### Mediation analysis

**Figure 5:**
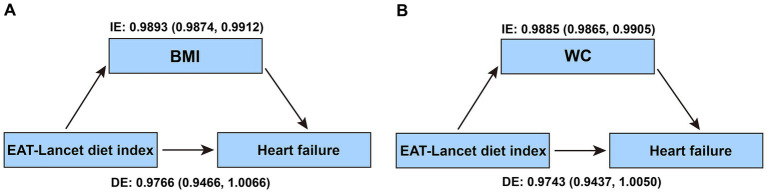
Mediation proportion of the association between EAT-Lancet diet index and heart failure risk mediated by BMI and WC.

As an exploratory analysis, we conducted a mediation analysis to examine the potential mediating roles of BMI and WC in the association between the EAT-Lancet diet index and HF risk. The results showed significant indirect effects through both BMI and WC, while the direct effects were not statistically significant, suggesting that BMI and WC may substantially mediate this association (Figure 5).

## Discussion

In this large-scale prospective cohort study from the UK Biobank, we found a significant inverse association between adherence to the EAT-Lancet diet pattern and the risk of HF events in individuals with CKMs stages 0–3. This association demonstrated a dose–response relationship and was validated through subgroup and sensitivity analyses. Notably, reduced intake of red meat (beef, lamb, and pork) and increased intake of peanuts or nuts were significantly inversely related to the risk of HF events. These findings suggest that optimizing the components of the EAT-Lancet diet may help reduce the risk of HF events in individuals with CKMs stages 0–3. Mediation analysis further suggested that BMI and WC may substantially mediate the relationship between the EAT-Lancet diet and HF outcomes. This highlights the importance of a healthy diet in the prevention of HF and suggests that a higher EAT-Lancet diet index can significantly reduce the risk of HF events in individuals with CKMs stages 0–3.

Many studies have demonstrated the association between adherence to the EAT-Lancet diet and various health outcomes, including diabetes, CVD, cancer, and all-cause mortality in the general population (12, 33–35). A study from the China Health and Nutrition Survey cohort found that a higher EAT-Lancet diet index was associated with a lower risk of mortality and CVD (12). Another study using the UK Biobank cohort also found that long-term adherence to a high EAT-Lancet diet index significantly reduced the occurrence of microvascular complications in patients with type 2 diabetes (21). However, these studies primarily focused on relatively healthy general populations or single-disease cohorts. There is still a lack of research on the relationship between the EAT-Lancet diet and the risk of HF in individuals with CKMs stages 0–3, as CKMs is a multi-system chronic condition. Individuals in stages 0–3 often have co-existing hypertension, diabetes, lipid metabolism abnormalities, and renal impairment (1, 36), which increases the overall risk of HF. They face not only the threat of single diseases but also the compounded health risks resulting from multiple disease interactions. In our study, we constructed the EAT-Lancet diet index using dietary questionnaire data from participants who completed at least two dietary assessments. We are the first to investigate the association between the EAT-Lancet diet index and the risk of HF in individuals with CKMs stages 0–3. We found that, compared to those with a low EAT-Lancet diet index, individuals in the high EAT-Lancet diet index group had a 12% lower risk of developing HF. Furthermore, there was a significant negative linear relationship between the EAT-Lancet diet index and the risk of HF in individuals with CKMs stages 0–3. Our study provides novel evidence for the role of the EAT-Lancet diet in reducing the risk of incident HF in individuals with CKMs stages 0–3, emphasizing the importance of maintaining a high EAT-Lancet diet index to lower the risk of HF in this population.

It is noteworthy that we further explored the associations between different components of the EAT-Lancet diet and HF risk. The results showed that reduced intake of red meat (beef, lamb, and pork) and increased intake of peanuts or nuts were significantly inversely associated with HF risk, which warrants further investigation. As is well known, red meat is a major source of protein and fat, and its relationship with CVD and diabetes has garnered widespread attention (37). Our findings are consistent with other studies. A meta-analysis of prospective cohort studies showed that consuming 100 grams of unprocessed red meat per day was associated with a 19% increased risk of developing type 2 diabetes (38, 39). High red meat intake is closely related to cardiovascular risk factors, such as inflammation, dyslipidemia, and atherosclerosis. Therefore, reducing red meat consumption is crucial for the prevention of HF risk in individuals with CKMs stages 0–3. On the other hand, peanuts and nuts are rich in healthy fats, dietary fiber, plant sterols, and antioxidants, and have been shown to offer multiple benefits, including anti-inflammatory effects, lipid-lowering properties, and improved blood glucose control (40–43). These foods play an important role in reducing the risk of CVD and HF. Therefore, it is recommended that individuals with CKMs stages 0–3 adopt a plant-based EAT-Lancet diet pattern, increasing the intake of nutrient-dense healthy foods like peanuts and nuts while reducing red meat consumption to effectively prevent and manage HF risk. However, the lack of association observed for other EAT-Lancet components does not necessarily imply they have no potential effect, as these dietary factors may exert influence through synergistic effects between different dietary components (44).

Subgroup analysis revealed a significant interaction between education level and the relationship between EAT-Lancet diet adherence and HF risk (*P*_interaction_ = 0.0487). Compared to individuals with lower education levels, participants with higher education levels who strictly adhered to the EAT-Lancet diet had a lower risk of developing HF. This result suggests that the protective effect of adherence to the EAT-Lancet diet is more pronounced among individuals with higher education levels. This may be related to the fact that individuals with higher education are more likely to have greater health awareness and easier access to healthcare services (45). Additionally, individuals with higher education often possess better information-seeking abilities, allowing them to more effectively follow health dietary guidelines, thereby reducing the risk of HF.

The pathophysiological mechanisms underlying the inverse association between the EAT-Lancet diet and HF risk in individuals with CKMs stages 0–3 remain unclear. Specifically, in CKMs stages 1–3, factors such as metabolic disorders, insulin resistance, obesity, and chronic inflammation gradually intensify. The EAT-Lancet diet may reduce HF risk by improving lipid metabolism (24), alleviating inflammation (46), and regulating blood glucose (47), which can effectively reduce the cardiac burden. Additionally, the results of two prospective cohort studies from Sweden suggest that adherence to a diet with high anti-inflammatory potential is associated with a lower incidence of HF (48), further supporting this notion. Our mediation analysis suggests that BMI and WC may substantially mediate the relationship between the EAT-Lancet diet index and HF events. This suggests that adherence to the EAT-Lancet diet may be associated with a lower risk of HF, potentially through pathways related to weight management and abdominal adiposity. This may be particularly relevant among individuals with CKMs stages 0–3, as obesity and excessive abdominal fat are risk factors for HF (49). These mediating factors may help explain the potential mechanisms through which dietary improvements contribute to HF prevention. Furthermore, the antioxidant components of high plant-based foods in the EAT-Lancet diet (50, 51)may help mitigate oxidative damage to the heart, further reducing the risk of HF. Although these mechanisms may vary across different stages of disease, the cardiovascular benefits of the EAT-Lancet diet are multifaceted. However, the specific biological mechanisms remain to be elucidated through further research, particularly to explore the long-term effects of the EAT-Lancet diet on gene expression, metabolic biomarkers, inflammatory responses, and cardiovascular health in individuals with CKMs stages 0–3.

The main strength of this study lies in its large cohort size and long follow-up period, which enabled us to conduct a robust analysis of the EAT-Lancet diet profile. However, there are several limitations. First, adherence to the diet was assessed through self-reported data, which may introduce reporting bias, as participants may either overestimate or underestimate their adherence to dietary habits. In addition, dietary assessment relies on 24-h recall methods, which may be subject to recall bias and fail to adequately reflect long-term dietary habits. However, the dietary assessment metrics used in our primary analysis have been demonstrated to correlate with repeatedly measured dietary indices (52). Second, it should be noted that the Knuppel score, as a binary scoring system, may not fully capture the complexity of dietary patterns. Individuals with similar scores may have substantially different dietary compositions. For example, a high score could reflect a diet rich in fruits, vegetables, and nuts but lacking other recommended components such as fish or legumes, while a lower score may still include beneficial foods alongside higher intakes of less healthy items. This heterogeneity within score categories may have influenced our findings and should be considered when interpreting the results. In addition, mediation analyses involving body mass index and waist circumference rely on strong, largely untestable assumptions in observational studies, including appropriate temporal ordering and the absence of unmeasured confounding, which may limit causal inference. Finally, the UK Biobank did not recruit participants older than 73 years, which may limit the generalizability of the findings to older populations. In addition, most participants were of European ancestry, and the observed associations may vary across populations with different ethnic backgrounds, cultural contexts, and socioeconomic conditions. Further studies are therefore needed to confirm the generalizability of these findings in more diverse populations.

## Conclusion

In conclusion, this study found a significant inverse association between adherence to the EAT-Lancet diet and the risk of HF among individuals with CKMs stages 0–3, particularly through reduced consumption of red meat and increased intake of peanuts or other nuts. Adherence to this dietary pattern may confer greater preventive benefits against HF in this population. These findings underscore the potential of individualized dietary interventions in enhancing HF prevention and provide scientific evidence to inform public health strategies.

## Data Availability

The raw data supporting the conclusions of this article will be made available by the authors, without undue reservation.

## References

[1] Ndumele CE Rangaswami J Chow SL Neeland IJ Tuttle KR Khan SS . Cardiovascular-kidney-metabolic health: a presidential advisory from the American Heart Association. Circulation. (2023) 148:1606–35. doi: 10.1161/CIR.0000000000001184 37807924

[2] Chen T Yin H Zhou Y Liang M. Relationship between estimated pulse wave velocity trajectories and cardiovascular disease risk in patients with cardiovascular-kidney-metabolic syndrome stages 0-3. Nutr Metab Cardiovasc Dis. (2025) 35:104192. doi: 10.1016/j.numecd.2025.104192 40615311

[3] Claudel SE Schmidt IM Waikar SS Verma A. Cumulative incidence of mortality associated with cardiovascular-kidney-metabolic (CKM) syndrome. J Am Soc Nephrol. (2025) 36:1343–51. doi: 10.1681/ASN.0000000637, 39932805 PMC12187233

[4] Marassi M Fadini GP. The cardio-renal-metabolic connection: a review of the evidence. Cardiovasc Diabetol. (2023) 22:195. doi: 10.1186/s12933-023-01937-x, 37525273 PMC10391899

[5] Kadowaki T Maegawa H Watada H Yabe D Node K Murohara T . Interconnection between cardiovascular, renal and metabolic disorders: a narrative review with a focus on Japan. Diabetes Obes Metab. (2022) 24:2283–96. doi: 10.1111/dom.14829, 35929483 PMC9804928

[6] Khan SS Coresh J Pencina MJ Ndumele CE Rangaswami J Chow SL . Novel prediction equations for absolute risk assessment of Total cardiovascular disease incorporating cardiovascular-kidney-metabolic health: a scientific statement from the American Heart Association. Circulation. (2023) 148:1982–2004. doi: 10.1161/CIR.0000000000001191, 37947094

[7] Bragazzi NL Zhong W Shu J Abu Much A Lotan D Grupper A . Burden of heart failure and underlying causes in 195 countries and territories from 1990 to 2017. Eur J Prev Cardiol. (2021) 28:1682–90. doi: 10.1093/eurjpc/zwaa147, 33571994

[8] Savarese G Becher PM Lund LH Seferovic P Rosano GMC Coats AJS. Global burden of heart failure: a comprehensive and updated review of epidemiology. Cardiovasc Res. (2023) 118:3272–87. doi: 10.1093/cvr/cvac013, 35150240

[9] Jia G Whaley-Connell A Sowers JR. Diabetic cardiomyopathy: a hyperglycaemia- and insulin-resistance-induced heart disease. Diabetologia. (2018) 61:21–8. doi: 10.1007/s00125-017-4390-4, 28776083 PMC5720913

[10] Lichtenstein AH Appel LJ Vadiveloo M Hu FB Kris-Etherton PM Rebholz CM . 2021 dietary guidance to improve cardiovascular health: a scientific statement from the American Heart Association. Circulation. (2021) 144:e472–87. doi: 10.1161/CIR.0000000000001031, 34724806

[11] Zhang S Marken I Stubbendorff A Ericson U Qi L Sonestedt E . The EAT-lancet diet index, plasma proteins, and risk of heart failure in a population-based cohort. JACC Heart Fail. (2024) 12:1197–208. doi: 10.1016/j.jchf.2024.02.017, 38573265

[12] Willett W Rockstrom J Loken B Springmann M Lang T Vermeulen S . Food in the Anthropocene: the EAT-lancet commission on healthy diets from sustainable food systems. Lancet. (2019) 393:447–92. doi: 10.1016/S0140-6736(18)31788-4, 30660336

[13] Stubbendorff A Sonestedt E Ramne S Drake I Hallstrom E Ericson U. Development of an EAT-lancet index and its relation to mortality in a Swedish population. Am J Clin Nutr. (2022) 115:705–16. doi: 10.1093/ajcn/nqab369, 34791011 PMC8895215

[14] Berthy F Toujgani H Duquenne P Fezeu LK Lairon D Pointereau P . Prospective association of the EAT-lancet reference diet with body weight changes and incidence of overweight and obesity in a French cohort. Am J Clin Nutr. (2025) 122:450–9. doi: 10.1016/j.ajcnut.2025.06.013, 40553762

[15] Lin X Wang S Huang J. The association between the EAT-lancet diet and diabetes: a systematic review. Nutrients. (2023) 15:15. doi: 10.3390/nu15204462 PMC10610026 37892537

[16] Sudlow C Gallacher J Allen N Beral V Burton P Danesh J . UK biobank: an open access resource for identifying the causes of a wide range of complex diseases of middle and old age. PLoS Med. (2015) 12:e1001779. doi: 10.1371/journal.pmed.1001779, 25826379 PMC4380465

[17] Allen NE Lacey B Lawlor DA Pell JP Gallacher J Smeeth L . Prospective study design and data analysis in UK biobank. Sci Transl Med. (2024) 16:eadf4428. doi: 10.1126/scitranslmed.adf4428, 38198570 PMC11127744

[18] Liu B Young H Crowe FL Benson VS Spencer EA Key TJ . Development and evaluation of the Oxford WebQ, a low-cost, web-based method for assessment of previous 24 h dietary intakes in large-scale prospective studies. Public Health Nutr. (2011) 14:1998–2005. doi: 10.1017/S1368980011000942, 21729481

[19] Liu D Li ZH Shen D Zhang PD Song WQ Zhang WT . Association of Sugar-Sweetened, artificially sweetened, and unsweetened coffee consumption with all-cause and cause-specific mortality: a large prospective cohort study. Ann Intern Med. (2022) 175:909–17. doi: 10.7326/M21-2977, 35635846

[20] Galante J Adamska L Young A Young H Littlejohns TJ Gallacher J . The acceptability of repeat internet-based hybrid diet assessment of previous 24-h dietary intake: administration of the Oxford WebQ in UK biobank. Br J Nutr. (2016) 115:681–6. doi: 10.1017/S0007114515004821, 26652593

[21] Zhang Z Huang Z Xi Y Han S Ye X Zhu H . Association between adherence to the EAT-lancet diet and risk of microvascular complications in type 2 diabetes: a cohort study. Diabetes Obes Metab. (2025) 27:3858–68. doi: 10.1111/dom.16414, 40269451

[22] Ye YX Chen JX Li Y Lai YW Lu Q Xia PF . Adherence to a planetary health diet, genetic susceptibility, and incident cardiovascular disease: a prospective cohort study from the UK biobank. Am J Clin Nutr. (2024) 120:648–55. doi: 10.1016/j.ajcnut.2024.06.014, 38950778

[23] Ho FK Gray SR Welsh P Petermann-Rocha F Foster H Waddell H . Associations of fat and carbohydrate intake with cardiovascular disease and mortality: prospective cohort study of UK biobank participants. BMJ. (2020) 368:m688. doi: 10.1136/bmj.m688 32188587 PMC7190059

[24] Zhang S Yan Y Zeng XF Gu Y Wu H Zhang Q . Associations of the EAT-lancet reference diet with metabolic dysfunction-associated steatotic liver disease and its severity: a multicohort study. Hepatology. (2025) 81:1583–94. doi: 10.1097/HEP.0000000000001039, 39094016

[25] Knuppel A Papier K Key TJ Travis RC. EAT-lancet score and major health outcomes: the EPIC-Oxford study. Lancet. (2019) 394:213–4. doi: 10.1016/S0140-6736(19)31236-X, 31235280

[26] Lu X Wu L Shao L Fan Y Pei Y Lu X . Adherence to the EAT-lancet diet and incident depression and anxiety. Nat Commun. (2024) 15:5599. doi: 10.1038/s41467-024-49653-8, 38961069 PMC11222463

[27] Stubbendorff A Stern D Ericson U Sonestedt E Hallstrom E Borne Y . A systematic evaluation of seven different scores representing the EAT-lancet reference diet and mortality, stroke, and greenhouse gas emissions in three cohorts. Lancet Planet Health. (2024) 8:e391–401. doi: 10.1016/S2542-5196(24)00094-9, 38849181

[28] Sterne JA White IR Carlin JB Spratt M Royston P Kenward MG . Multiple imputation for missing data in epidemiological and clinical research: potential and pitfalls. BMJ. (2009) 338:b2393. doi: 10.1136/bmj.b2393, 19564179 PMC2714692

[29] Geng T Xu W Gao H Zhang J Zou J Wang K . Relationship between control of cardiovascular risk factors and chronic kidney disease progression, cardiovascular disease events, and mortality in Chinese adults. J Am Coll Cardiol. (2024) 84:1313–24. doi: 10.1016/j.jacc.2024.06.041, 39322325

[30] Abbasi F Blasey C Reaven GM. Cardiometabolic risk factors and obesity: does it matter whether BMI or waist circumference is the index of obesity? Am J Clin Nutr. (2013) 98:637–40. doi: 10.3945/ajcn.112.047506 23885045 PMC3743728

[31] Gonzalez-Galvez N Ribeiro J Mota J. Metabolic syndrome and cardiorespiratory fitness in children and adolescents: the role of obesity as a mediator. J Pediatr Endocrinol Metab. (2021) 34:1031–9. doi: 10.1515/jpem-2020-0640, 34162024

[32] Lee YS Yang PS Jang E Kim D Yu HT Kim TH . Association between obesity and heart failure and related atrial fibrillation: patient-level data comparisons of two cohort studies. Yonsei Med J. (2024) 65:10–8. doi: 10.3349/ymj.2023.0264 38154475 PMC10774652

[33] Zhang S Stubbendorff A Olsson K Ericson U Niu K Qi L . Adherence to the EAT-lancet diet, genetic susceptibility, and risk of type 2 diabetes in Swedish adults. Metabolism. (2023) 141:155401. doi: 10.1016/j.metabol.2023.155401, 36682448

[34] Hu FL Liu JC Li DR Xu YL Liu BQ Chen X . EAT-lancet diet pattern, genetic risk, and risk of colorectal cancer: a prospective study from the UK biobank. Am J Clin Nutr. (2025) 121:1017–24. doi: 10.1016/j.ajcnut.2025.02.025 39993568

[35] Liu J Shen Q Wang X. Emerging EAT-lancet planetary health diet is associated with major cardiovascular diseases and all-cause mortality: a global systematic review and meta-analysis. Clin Nutr. (2024) 43:167–79. doi: 10.1016/j.clnu.2024.10.021, 39489999

[36] Ndumele CE Neeland IJ Tuttle KR Chow SL Mathew RO Khan SS . A synopsis of the evidence for the science and clinical Management of Cardiovascular-Kidney-Metabolic (CKM) syndrome: a scientific statement from the American Heart Association. Circulation. (2023) 148:1636–64. doi: 10.1161/CIR.0000000000001186, 37807920

[37] Micha R Wallace SK Mozaffarian D. Red and processed meat consumption and risk of incident coronary heart disease, stroke, and diabetes mellitus: a systematic review and meta-analysis. Circulation. (2010) 121:2271–83. doi: 10.1161/CIRCULATIONAHA.109.924977, 20479151 PMC2885952

[38] Micha R Michas G Mozaffarian D. Unprocessed red and processed meats and risk of coronary artery disease and type 2 diabetes--an updated review of the evidence. Curr Atheroscler Rep. (2012) 14:515–24. doi: 10.1007/s11883-012-0282-8, 23001745 PMC3483430

[39] Pan A Sun Q Bernstein AM Schulze MB Manson JE Willett WC . Red meat consumption and risk of type 2 diabetes: 3 cohorts of US adults and an updated meta-analysis. Am J Clin Nutr. (2011) 94:1088–96. doi: 10.3945/ajcn.111.018978, 21831992 PMC3173026

[40] Jackson CL Hu FB. Long-term associations of nut consumption with body weight and obesity. Am J Clin Nutr. (2014) 100:408S–11S. doi: 10.3945/ajcn.113.071332, 24898229 PMC4144111

[41] Mohammadifard N Salehi-Abargouei A Salas-Salvado J Guasch-Ferre M Humphries K Sarrafzadegan N. The effect of tree nut, peanut, and soy nut consumption on blood pressure: a systematic review and meta-analysis of randomized controlled clinical trials. Am J Clin Nutr. (2015) 101:966–82. doi: 10.3945/ajcn.114.091595, 25809855

[42] Viguiliouk E Kendall CW Blanco Mejia S Cozma AI Ha V Mirrahimi A . Effect of tree nuts on glycemic control in diabetes: a systematic review and meta-analysis of randomized controlled dietary trials. PLoS One. (2014) 9:e103376. doi: 10.1371/journal.pone.0103376, 25076495 PMC4116170

[43] Mikail MA Ahmed IA Ibrahim M Hazali N Abdul Rasad MS Abdul Ghani R . Baccaurea angulata fruit inhibits lipid peroxidation and induces the increase in antioxidant enzyme activities. Eur J Nutr. (2016) 55:1435–44. doi: 10.1007/s00394-015-0961-7, 26091909

[44] Tapsell LC Neale EP Satija A Hu FB. Foods, nutrients, and dietary patterns: interconnections and implications for dietary guidelines. Adv Nutr. (2016) 7:445–54. doi: 10.3945/an.115.011718, 27184272 PMC4863273

[45] Pengpid S Peltzer K. Chronic conditions, multimorbidity, and quality of life among patients attending monk healers and primary care clinics in Thailand. Health Qual Life Outcomes. (2021) 19:61. doi: 10.1186/s12955-021-01707-x, 33622328 PMC7903786

[46] Feng J Pan C Gou Y Yang X Cheng S Wei W . EAT-lancet diet modifies the risk of rheumatoid arthritis through metabolomic signature. Arthritis Rheumatol. (2026) 78:68–79. doi: 10.1002/art.43297, 40546003

[47] Karabicak M Yuksel A. EAT-lancet diet adherence in patients with type 2 diabetes: a cross-sectional study. Food Sci Nutr. (2025) 13:e70650. doi: 10.1002/fsn3.70650, 40688606 PMC12271971

[48] Kaluza J Levitan EB Michaelsson K Wolk A. Anti-inflammatory diet and risk of heart failure: two prospective cohort studies. Eur J Heart Fail. (2020) 22:676–82. doi: 10.1002/ejhf.1746, 31975476

[49] Wang B Yang X Wei B Ren T Huang N Escobar C . Associations between waist circumference, central obesity, and the presence of non-valvular atrial fibrillation patients with heart failure. J Thorac Dis. (2024) 16:2049–59. doi: 10.21037/jtd-24-170, 38617752 PMC11009574

[50] Jomova K Raptova R Alomar SY Alwasel SH Nepovimova E Kuca K . Reactive oxygen species, toxicity, oxidative stress, and antioxidants: chronic diseases and aging. Arch Toxicol. (2023) 97:2499–574. doi: 10.1007/s00204-023-03562-9, 37597078 PMC10475008

[51] Shanmugasundaram K Block K. Renal carcinogenesis, tumor heterogeneity, and reactive oxygen species: tactics evolved. Antioxid Redox Signal. (2016) 25:685–701. doi: 10.1089/ars.2015.6569, 27287984 PMC5069729

[52] Heianza Y Zhou T Sun D Hu FB Qi L. Healthful plant-based dietary patterns, genetic risk of obesity, and cardiovascular risk in the UK biobank study. Clin Nutr. (2021) 40:4694–701. doi: 10.1016/j.clnu.2021.06.018, 34237696 PMC8338907

