# Supplementary material for: Adherence to the EAT-Lancet diet and the risk of heart failure in individuals with cardiovascular-kidney-metabolic syndrome stages 0–3: A prospective cohort study
Source: Front Nutr. 2026 May 13;13:1802388. doi: 10.3389/fnut.2026.1802388 (PMC13212115; doi:10.3389/fnut.2026.1802388)

Supplemental Material

**CKM Syndrome Stages Definition^1^**

**Stage 0:** Participants classified as CKM Stage 0 were those who did not meet criteria for any of the higher stages and had a normal BMI—defined as <23 kg/m² for Asian individuals and <25 kg/m² for individuals of other racial and ethnic backgrounds—as well as normal waist circumference, defined as <80 cm for Asian women and <90 cm for Asian men, and <88 cm and <102 cm for women and men in other racial and ethnic groups, respectively.

**Stage 1**: CKM Stage 1 included individuals exhibiting signs of excess and/or dysfunctional adiposity, as indicated by either elevated BMI (≥23 kg/m² for Asian; >25 kg/m² for other racial and ethnic groups), increased waist circumference (≥80 cm for Asian women and ≥90 cm for Asian men; ≥88 cm and ≥102 cm for women and men in other racial and ethnic groups, respectively), or prediabetes, defined as glycated hemoglobin between 5.7% and <6.5% or fasting blood glucose ranging from 100 to 126 mg/dL.

**Stage 2**: CKM Stage 2 comprised individuals presenting with either metabolic risk factors or moderate-to-high-risk chronic kidney disease (CKD), as defined by the Kidney Disease Improving Global Outcomes (KDIGO) guidelines and endorsed by the American Heart Association. Metabolic risk factors included elevated fasting triglycerides (≥135 mg/dL), hypertension, diabetes, or the presence of metabolic syndrome—defined as meeting at least 3 of the following: increased waist circumference, reduced HDL cholesterol levels (<40 mg/dL for men or <50 mg/dL for women), fasting triglycerides ≥150 mg/dL, elevated blood pressure (systolic ≥130 mmHg, diastolic ≥80 mmHg, or current use of antihypertensive medications), and prediabetes. CKD classification was determined by estimated glomerular filtration rate and urinary albumin-to-creatinine ratio.

**Stage 3**: CKM Stage 3 was defined by the presence of very-high-risk chronic kidney disease (CKD) stages according to KDIGO guidelines or a high predicted 10-year risk of cardiovascular disease (CVD), as estimated using the American Heart Association (AHA) PREVENT (Predicting Risk of CVD EVENTs) equations. High CVD risk was defined as a predicted 10-year risk ≥20%, following established thresholds (https://professional.heart.org/en/guidelines-and-statements/prevent-calculator).

The PREVENT equations were developed and validated for individuals aged 30 to 79 years. Given that CKM staging has no age restriction, individuals aged ≥79 years and those younger than 30 years were not excluded but were assigned an age of 79 and 30 years, respectively. In sensitivity analyses, participants outside the 30–79-year age range were excluded. The PREVENT models were designed for specific parameter ranges, including total cholesterol (130–320 mg/dL), HDL cholesterol (20–100 mg/dL), systolic blood pressure (90–200 mmHg), and glomerular filtration rate (GFR; 14–140 mL/min/1.73m²). To remain within these model specifications, values exceeding these thresholds were truncated to the nearest boundary. For example, a systolic blood pressure of 210 mmHg was recoded as 200 mmHg for CVD risk estimation.

1. Aggarwal R, Ostrominski JW and Vaduganathan M. Prevalence of Cardiovascular-Kidney-Metabolic Syndrome Stages in US Adults, 2011-2020. JAMA. 2024;331:1858-1860.

**Supplemental Tables**

**Table S1. Definition of portion size and food items used for the EAT-Lancet diet index**

**Table S2. Cut-offs for each component of the EAT-Lancet diet index**

**Table S3. Subgroup Analyses: Association Between EAT-Lancet Diet Index and Risk of Incident Heart Failure**

**Table S4. Sensitivity analysis excluding outcome events occurring within the two years of follow-up in CKMs stages 0-3 individuals**

**Table S5. Sensitivity analysis excluding Individuals with missing covariates in CKMs stages 0-3 individuals**

**Table S6. Sensitivity analysis of competing risk model**

**Table S7. Sensitivity analysis restricted to individuals with CKMs stages 1-3**

**Table S8. Sensitivity analysis with additional adjustment for cardiometabolic factors**

**Supplemental Figures**

**Figure S1. Distribution of EAT-Lancet diet index across CKMs stages 0-3 individuals**

**Table S1. Definition of portion size and food items used for the EAT-Lancet diet index**

| Diet component | Food items based on the Oxford WebQ questionnaire | Portion size (g) |
| --- | --- | --- |
| **Whole grains** |  |  |
| 1 Rice, wheat, corn, and other | Porridge | 203.5 |
|  | Muesli, oat crunch | 80 |
|  | Sweetened cereal | 38 |
|  | Plain cereal | 30 |
|  | Bran cereal | 50 |
|  | Whole-wheat cereal, other cereal | 44 |
|  | White pasta, wholemeal pasta | 230 |
|  | White rice | 175 |
|  | Brown rice | 180 |
|  | Sushi | 278 |
|  | Snackpot | 280 |
|  | Couscous | 150 |
|  | Other grain | 157 |
| **Tubers and starchy vegetables** |  |  |
| 2 Potatoes | Sweet potato intake | 130 |
|  | Mashed potato intake | 180 |
|  | Boiled/baked potatoes intake | 175 |
|  | Fried potatoes intake | 180 |
| **Vegetables** |  |  |
| 3 All vegetables | Quorn, mixed vegetable, vegetable pieces, coleslaw, side salad, avocado, broad bean, green bean, beetroot, broccoli, butternut squash, cabbage, carrot, cauliflower, celery, courgette, cucumber, garlic, leek, lettuce, mushroom, onion, parsnip, pea, sweet pepper, spinach, fresh tomato, tinned tomato, turnip, watercress, other vegetable | 100 |
|  | Mixed vegetable, vegetable pieces | 90 |
|  | Coleslaw, side salad | 66 |
|  | Leek | 80 |
|  | Lettuce | 35 |
|  | Mushroom | 44 |
|  | Parsnip, pea | 65 |
|  | Sweet pepper | 160 |
|  | Spinach | 90 |
|  | Fresh tomato | 85 |
|  | Tinned tomato | 135 |
|  | Turnip | 60 |
|  | Watercress | 20 |
|  | Sweetcorn | 43 |
| **Fruits** |  |  |
| 4 All fruits | Stewed fruit | 140 |
|  | Prune, dried fruit, other fruit | 60 |
|  | Mixed fruit | 105 |
|  | Apple, banana, orange | 120 |
|  | Berry | 40 |
|  | Cherry | 34 |
|  | Grapefruit | 148 |
|  | Grape | 100 |
|  | Mango | 113 |
|  | Melon | 180 |
|  | Satsuma | 70 |
|  | Peach | 150 |
|  | Pear | 160 |
|  | Pineapple | 80 |
|  | Plum | 55 |
| **Dairy foods** |  |  |
| 5 Whole milk derivative equivalents | Milk | 259 |
|  | Favoured milk | 250 |
|  | Yogurt | 125 |
|  | Low fat hard cheese, hard cheese, soft cheese, feta, mozzarella, goat ’s cheese, other cheese, low fat hard cheese | 40 |
|  | Blue cheese | 35 |
|  | Low fat cheese spread, cheese spread | 15 |
|  | Cottage cheese | 60 |
| **Protein sources** |  |  |
| 6 Beef, lamb, pork | Bacon | 46 |
|  | Ham | 23 |
|  | Liver | 70 |
|  | Sausage | 30 |
|  | Beef, pork, lamb | 120 |
| 7 Chicken, other poultry | Crumbed or deep-fried poultry | 100 |
|  | Poultry | 130 |
| 8 Eggs | Whole egg | 50 |
|  | Omelette, eggs in sandwich, scotch egg | 120 |
|  | Other egg | 75 |
| 9 Fish | Shellfish | 40 |
|  | Tinned tuna | 92 |
|  | Oily fish, breaded fish, other fish | 100 |
|  | Battered fish | 150 |
|  | White fish | 120 |
| **Legumes** |  |  |
| 10 Dry beans, lentils, peas | Pulses intakes | 70 |
|  | Bean (baked beans) | 135 |
|  | Pea | 65 |
| 11 Soy foods | Tofu | 90 |
| 12 Peanuts or tree nuts | Unsalted peanuts | 40 |
|  | Salted nuts | 40 |
|  | Unsalted nuts | 40 |
|  | Salted nuts and seeds | - |
| **Added fats** |  |  |
| 13 Palm oil, unsaturated oils, dairy fats (incl. in milk), lard or tallow | Saturated fat | - |
| Unsaturated-to-saturated fat ratio | Polyunsaturated fat | - |
| **Added sugars** |  |  |
| 14 All sweeteners | Free sugar | - |

**Table S2. Cut-offs for each component of the EAT-Lancet diet index**

| Diet component | Criteria for score of 1 | Criteria for score of 0 |
| --- | --- | --- |
| **Whole grains** |  |  |
| 1 Rice, wheat, corn, and other | ≤ 464 g/day | >464 g/day |
| **Tubers and starchy vegetables** |  |  |
| 2 Potatoes | ≤100 g/day | >100 g/day |
| **Vegetables** |  |  |
| 3 All vegetables | ≥200 g/day | <200 g/day |
| **Fruits** |  |  |
| 4 All fruits | ≥100 g/day | <100 g/day |
| **Dairy foods** |  |  |
| 5 Whole milk derivative equivalents | ≤500 g/day | >500 g/day |
| **Protein sources** |  |  |
| 6 Beef, lamb, pork | ≤28 g/day | >28 g/day |
| 7 Chicken, other poultry | ≤58 g/day | >58 g/day |
| 8 Eggs | ≤25 g/day | >25 g/day |
| 9 Fish | ≤100 g/day | >100g/day |
| **Legumes** |  |  |
| 10 Dry beans, lentils, peas | ≤100 g/day | >100 g/day |
| 11 Soy foods | ≤50 g/day | >50 g/day |
| 12 Peanuts or tree nuts | ≥25 g/day | <25 g/day |
| **Added fats** |  |  |
| 13 Palm oil, unsaturated oils, dairy fats (incl. in milk), lard or tallow |  |  |
| Ratio of unsaturated to saturated fat intake | ≥0.8 | <0.8 |
| **Added sugars** |  |  |
| 14 All sweeteners | ≤31g/day | >31g/day |

Note: A score of 1 point was assigned to each food component that met the recommended criteria of the EAT-Lancet diet pattern. The maximum possible score was 14, with a minimum score of 0.

**Table S3. Subgroup Analyses: Association Between EAT-Lancet Diet Index and Risk of Incident Heart Failure**

|  | **EAT-Lancet Diet Index** | | |  |
| --- | --- | --- | --- | --- |
| **Subgroups** | **Low ( < 8)** | **Moderate ( = 8)** | **High ( > 8)** | ***P* for interation** |
| **Age** |  |  |  | 0.534 |
| Age<65 |  |  |  |  |
| Event/Total | 473/30,410 | 452/30,146 | 573/42,312 | _ |
|  | 1.00 | 0.99 (0.87, 1.13) | 0.94 (0.83, 1.06) | _ |
| Age≥65 |  |  |  |  |
| Event/Total | 337/5,673 | 303/5,456 | 316/6,852 | _ |
|  | 1.00 | 0.93 (0.80, 1.09) | 0.79 (0.67, 0.92) | _ |
| **Sex** |  |  |  | 0.662 |
| Male |  |  |  |  |
| Event/Total | 518/16,349 | 456/15,567 | 534/20,298 | _ |
|  | 1.00 | 0.94 (0.83, 1.06) | 0.88 (0.78, 0.99) | _ |
| Female |  |  |  |  |
| Event/Total | 292/19,734 | 299/20,035 | 355/28,866 | _ |
|  | 1.00 | 1.02 (0.87, 1.20) | 0.89 (0.76, 1.04) | _ |
| **Education** |  |  |  | 0.049 |
| College or university |  |  |  |  |
| Event/Total | 117/5,383 | 85/5,686 | 141/8,241 | _ |
|  | 1.00 | 0.74 (0.56, 0.98) | 0.89 (0.70, 1.14) | _ |
| Others |  |  |  |  |
| Event/Total | 693/30,700 | 670/29,946 | 748/40,923 | _ |
|  | 1.00 | 1.01 (0.91, 1.13) | 0.87 (0.80, 0.98) | _ |
| **Current smoker** |  |  |  | 0.800 |
| Yes |  |  |  |  |
| Event/Total | 729/33,605 | 674/33,098 | 800/45,762 | _ |
|  | 1.00 | 0.96 (0.86, 1.06) | 0.87 (0.79, 0.96) | _ |
| No |  |  |  |  |
| Event/Total | 81/2,478 | 81/2,504 | 89/3.402 | _ |
|  | 1.00 | 1.04 (0.76, 1.42) | 0.92 (0.68, 1.25) | _ |
| **Current drinker** |  |  |  | 0.801 |
| Yes |  |  |  |  |
| Event/Total | 66/1,882 | 58/1,901 | 80/3,081 | _ |
|  | 1.00 | 0.94 (0.66, 1.34) | 0.82 (0.58, 1.13) | _ |
| No |  |  |  |  |
| Event/Total | 744/34,201 | 697/33,701 | 809/46,083 | _ |
|  | 1.00 | 0.97 (0.87, 1.08) | 0.88 (0.80, 0.97) | _ |

Note: EAT-Lancet index was categorized as low ( < 8), moderate ( = 8), and high ( > 8). The Cox proportional hazards models were adjusted for age, sex, current drinker, current smoker, education, income, Ethnicity, Townsend deprivation index, Family history of CVD.

**Table S4. Sensitivity analysis excluding outcome events occurring within the two years of follow-up in CKMs stages 0-3 individuals**

|  | **EAT-Lancet diet index** | | |  |  |
| --- | --- | --- | --- | --- | --- |
|  | **Low ( < 8)** | **Moderate ( = 8)** | **High ( > 8)** | **Per 1-SD increase** | ***P* for trend** |
| Event/Total | 782/36,055 | 727/35,574 | 852/49,127 | _ | _ |
| IR | 1.48 | 1.39 | 1.18 | _ | _ |
|  | 1.00 | 0.97 (0.87, 1.07) | 0.87 (0.79, 0.96) | 0.96 (0.92, 0.99) | 0.006 |

Note: EAT-Lancet index was categorized as low ( < 8), moderate ( = 8), and high ( > 8). The Cox proportional hazards models were adjusted for age, sex, current drinking, current smoking, education, income, Ethnicity, Townsend deprivation index, Family history of CVD.

CKMs, Cardiovascular-Kidney-Metabolic syndrome; IR, incidence rate (per 1000 person-years).

**Table S5. Sensitivity analysis excluding individuals with missing covariates in CKMs stages 0-3 individuals**

|  | **EAT-Lancet diet index** | | |  |  |
| --- | --- | --- | --- | --- | --- |
|  | **Low ( < 8)** | **Moderate ( = 8)** | **High ( > 8)** | **Per 1-SD increase** | ***P* for trend** |
| Event/Total | 693/31,885 | 646/31,307 | 747/43,215 | _ | _ |
| IR | 1.48 | 1.40 | 1.17 | _ | _ |
|  | 1.00 | 0.98 (0.88, 1.09) | 0.88 (0.79, 0.98) | 0.95 (0.91, 0.99) | 0.008 |

Note: EAT-Lancet index was categorized as low ( < 8), moderate ( = 8), and high ( > 8). The Cox proportional hazards models were adjusted for age, sex, current drinking, current smoking, education, income, Ethnicity, Townsend deprivation index, Family history of CVD.

CKMs, Cardiovascular-Kidney-Metabolic syndrome; IR, incidence rate (per 1000 person-years).

**Table S6. Sensitivity analysis of competing risk model**

|  | **EAT-Lancet diet index** | | |  |
| --- | --- | --- | --- | --- |
|  | **Low ( < 8)** | **Moderate ( = 8)** | **High ( > 8)** | ***P* for trend** |
| Event/Total | 4,104/36,083 | 4,001/35,602 | 5,251/49,164 | _ |
| IR | 7.77 | 7.64 | 7.23 | _ |
|  | 1.00 | 0.97 (0.88, 1.07) | 0.88 (0.80, 0.97) | 0.007 |

Note: EAT-Lancet index was categorized as low ( < 8), moderate ( = 8), and high ( > 8). The Cox proportional hazards models were adjusted for age, sex, current drinking, current smoking, education, income, Ethnicity, Townsend deprivation index, Family history of CVD.

**Table S7. Sensitivity analysis restricted to individuals with CKMs stages 1–3**

|  | **EAT-Lancet diet index** | | |  |  |
| --- | --- | --- | --- | --- | --- |
|  | **Low ( < 8)** | **Moderate ( = 8)** | **High ( > 8)** | **Per 1-SD increase** | ***P* for trend** |
| Event/Total | 734/28,187 | 693/27,398 | 792/36,601 | _ | _ |
| IR | 1.79 | 1.73 | 1.47 | _ | _ |
|  | 1.00 | 0.99 (0.89, 1.10) | 0.89 (0.80, 0.98) | 0.95 (0.91, 0.99) | 0.018 |

Note: EAT-Lancet index was categorized as low ( < 8), moderate ( = 8), and high ( > 8). The Cox proportional hazards models were adjusted for age, sex, current drinking, current smoking, education, income, Ethnicity, Townsend deprivation index, Family history of CVD.

CKMs, Cardiovascular-Kidney-Metabolic syndrome; IR, incidence rate (per 1000 person-years).

**Table S8. Sensitivity analysis with additional adjustment for cardiometabolic factors**

|  | **EAT-Lancet diet index** | | |  |  |
| --- | --- | --- | --- | --- | --- |
|  | **Low ( < 8)** | **Moderate ( = 8)** | **High ( > 8)** | **Per 1-SD increase** | ***P* for trend** |
| Event/Total | 810/36,083 | 755/35,602 | 889/49,164 | _ | _ |
| IR | 1.53 | 1.44 | 1.23 | _ | _ |
|  | 1.00 | 0.98 (0.94, 1.02) | 0.92 (0.88, 0.95) | 0.97 (0.95, 0.98) | <0.001 |

Note: EAT-Lancet index was categorized as low ( < 8), moderate ( = 8), and high ( > 8). The Cox proportional hazards models were adjusted for age, sex, current drinking, current smoking, education, income, Ethnicity, Townsend deprivation index, Family history of CVD, SBP, DBP, FBG, eGFR, HDL-C, LDL-C, WC, BMI.

SBP: systolic blood pressure; DBP: diastolic blood pressure; FBG: fasting blood glucose; eGFR: estimated glomerular filtration rate; HDL-C: high-density lipoprotein cholesterol; LDL-C: low-density lipoprotein cholesterol; WC: waist circumference; BMI: body mass index; IR, incidence rate (per 1000 person-years).

**Figure S1. Distribution of EAT-Lancet diet index across CKMs stages 0-3 individuals**


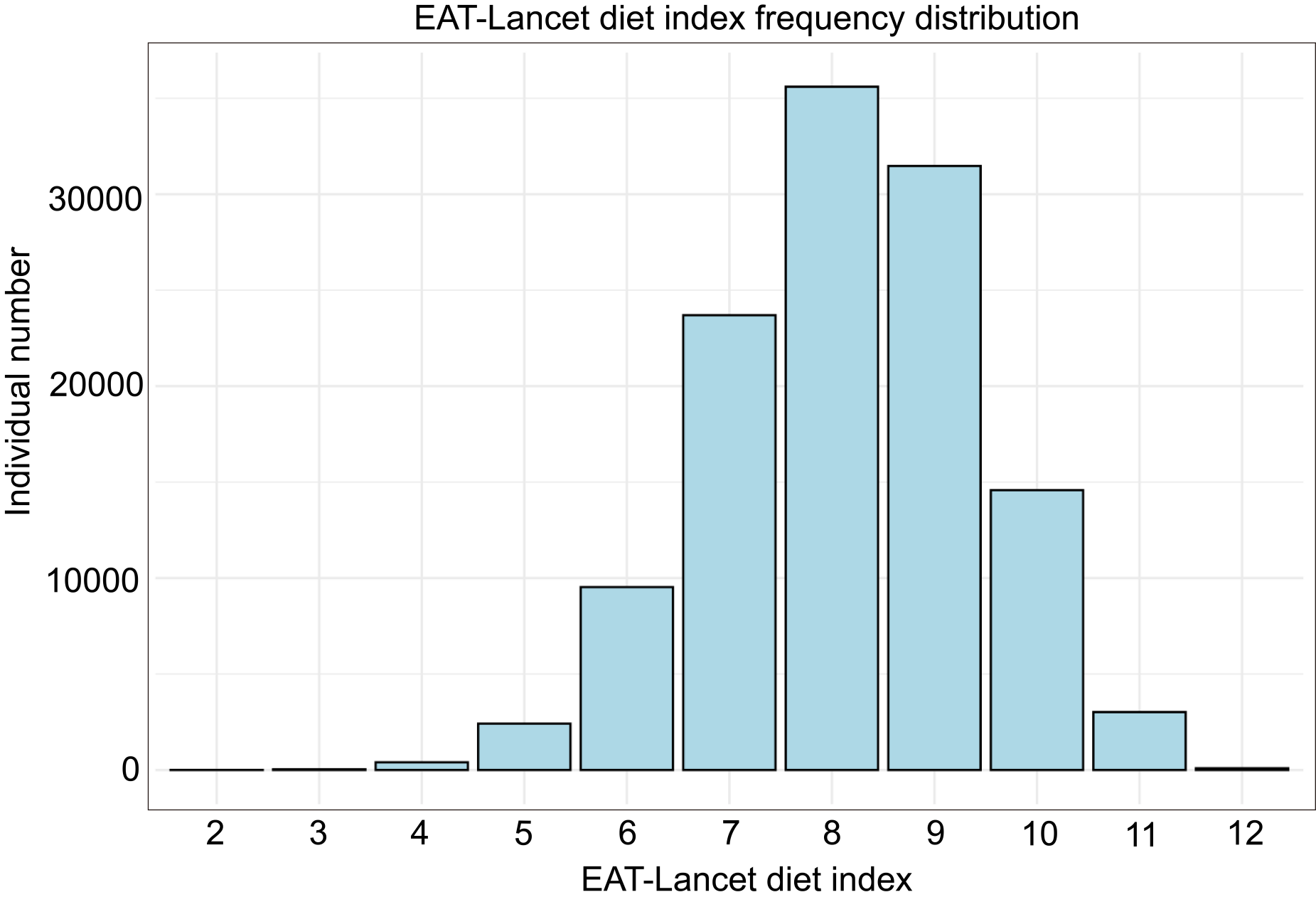

Supplement: Supplementary file 1 [file Table_1.docx]
